# Case Report: The importance of examining colon and rectum in patients with appendiceal cancer

**DOI:** 10.12688/f1000research.50909.2

**Published:** 2022-03-03

**Authors:** Hugin Reistrup, Siv Fonnes, Jacob Rosenberg, Kristoffer Andresen

**Affiliations:** 1 Center for Perioperative Optimization, Department of Surgery, Herlev and Gentofte Hospitals, University of Copenhagen, Herlev, DK-2730, Denmark

**Keywords:** Appendicitis, appendix cancer, appendiceal cancer, synchronous, rectum cancer, rectal cancer, digital rectal examination, case report

## Abstract

Appendiceal cancer is rare and is often diagnosed incidentally in patients undergoing appendectomy for acute appendicitis. However, patients with appendiceal cancer are at increased risk of synchronous malignancy. In this case report, we present a 58-year-old man initially diagnosed with acute appendicitis after presenting to the emergency department with abdominal pain. He had an appendectomy and was discharged the following day. Unexpectedly, the postoperative histopathologic examination showed a primary adenocarcinoma in the appendix. A computed tomography scan showed rectal wall thickening and the patient was referred to colonoscopy where an experienced endoscopist found a rectal tumor during the digital rectal examination prior to the colonoscopy. The tumor was initially missed by the newly qualified doctor who examined the patient during his first admittance to hospital. The patient’s two primary cancers were treated with a laparoscopic right hemicolectomy for the appendiceal cancer and a low anterior resection for the rectal cancer. This case supports the importance of a full colorectal workup in patients with appendiceal cancer. It also emphasizes the value of a thorough digital rectal examination and the need for improved focus on teaching and practice of the procedure.

## Introduction

Primary appendiceal neoplasms are rare and represent 1% of all gastrointestinal cancers, and the incidence is increasing ^1, 2^. Typically, symptoms are vague and the patient often presents with acute appendicitis with the tumor being diagnosed incidentally during histopathological examination after appendectomy. In other cases, tumors can present as bowel obstruction or as a palpable pelvic mass ^3^. Several studies have found that appendiceal neoplasms are associated with an increased risk of synchronous colorectal lesions ^1, 3– 6^. Multiple primary tumors can be divided into synchronous or metachronous tumors. Synchronous tumors are defined as tumors diagnosed less than six months apart while metachronous tumors are diagnosed more than six months apart ^7^. It has been suggested, that the association between synchronous tumors in the appendix and the colon and rectum could be due to similar histological pattern and that the appendix derives embryologically from the cecum ^4^. Guidelines recommend that patients with appendiceal neoplasms should undergo colonoscopy ^3^. Also, guidelines call for abdominal examination and a digital rectal examination (DRE) ^3^. DRE is a simple, quick, and inexpensive clinical procedure. One retrospective study found a sensitivity of 76% and specificity of 92% of the DRE for finding palpable rectal tumors ^8^. Although it is a useful clinical tool for diagnostics and screening, DRE has a learning curve and the sensitivity is highly examiner-dependent ^9^.

We present a patient with synchronous tumors in the appendix and rectum. This case underlines the importance of a full colorectal examination in patients diagnosed with primary appendiceal neoplasms and highlights the value of a thorough DRE. The case is presented in accordance with the CARE guideline ^10^.

## Case presentation

**Figure 1.**
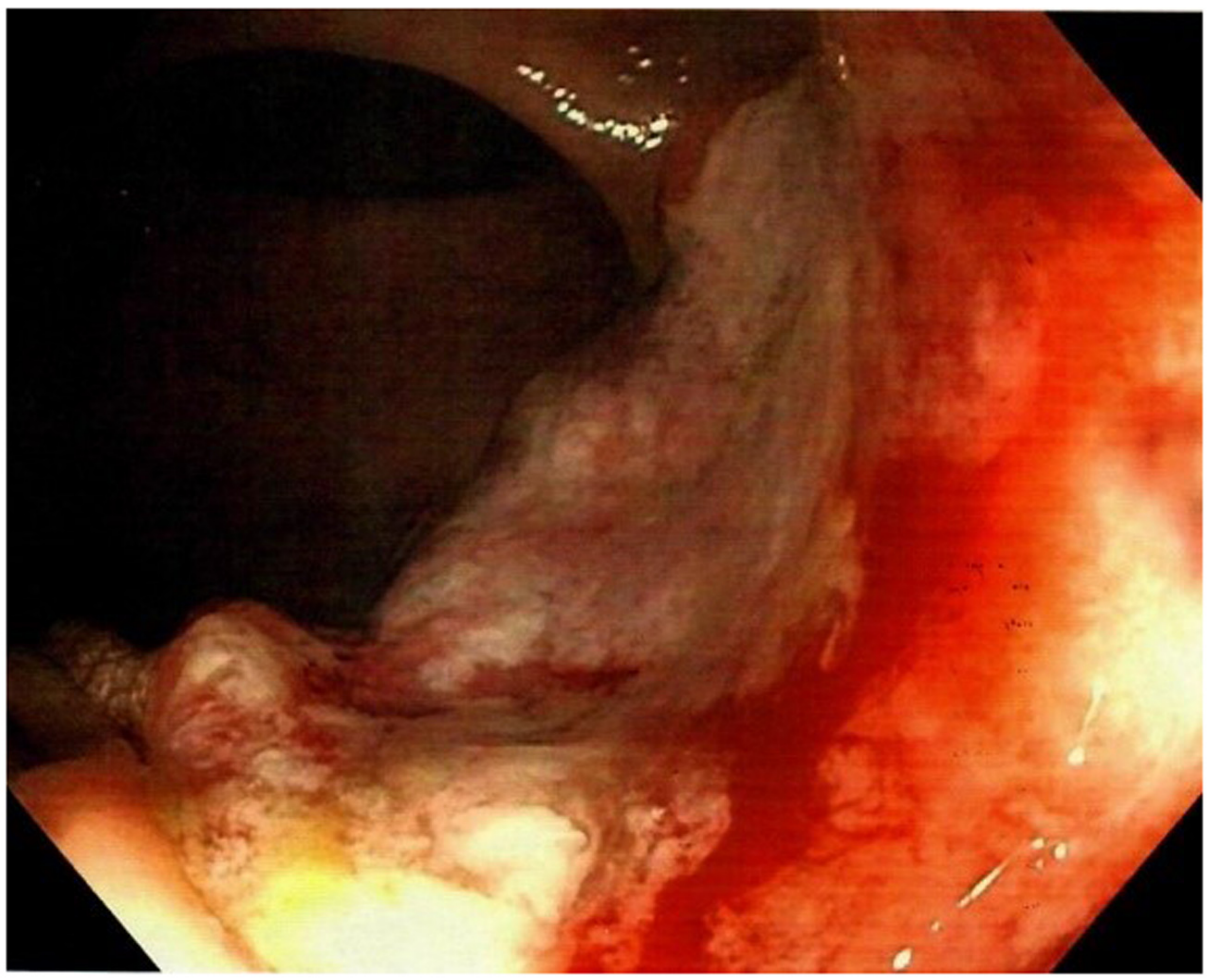
Colonoscopy showing the rectal tumor.

**Figure 2.**
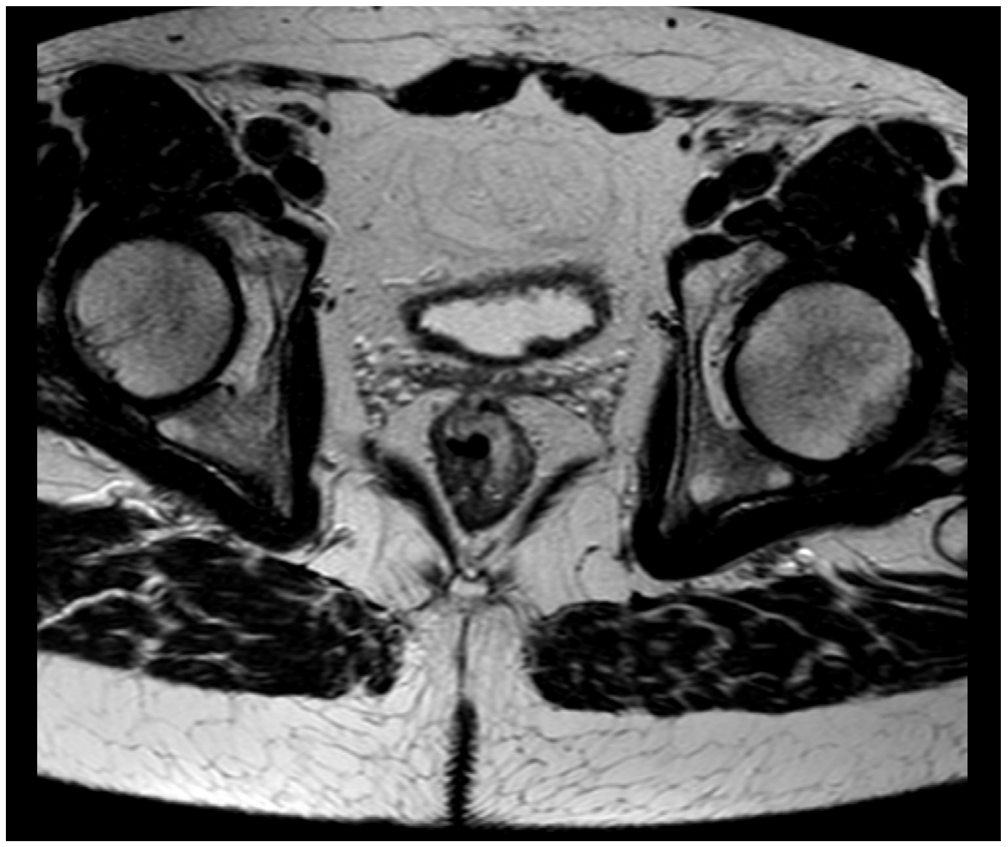
Magnetic resonance imaging scan showing the rectal tumor.

**Figure 3.**
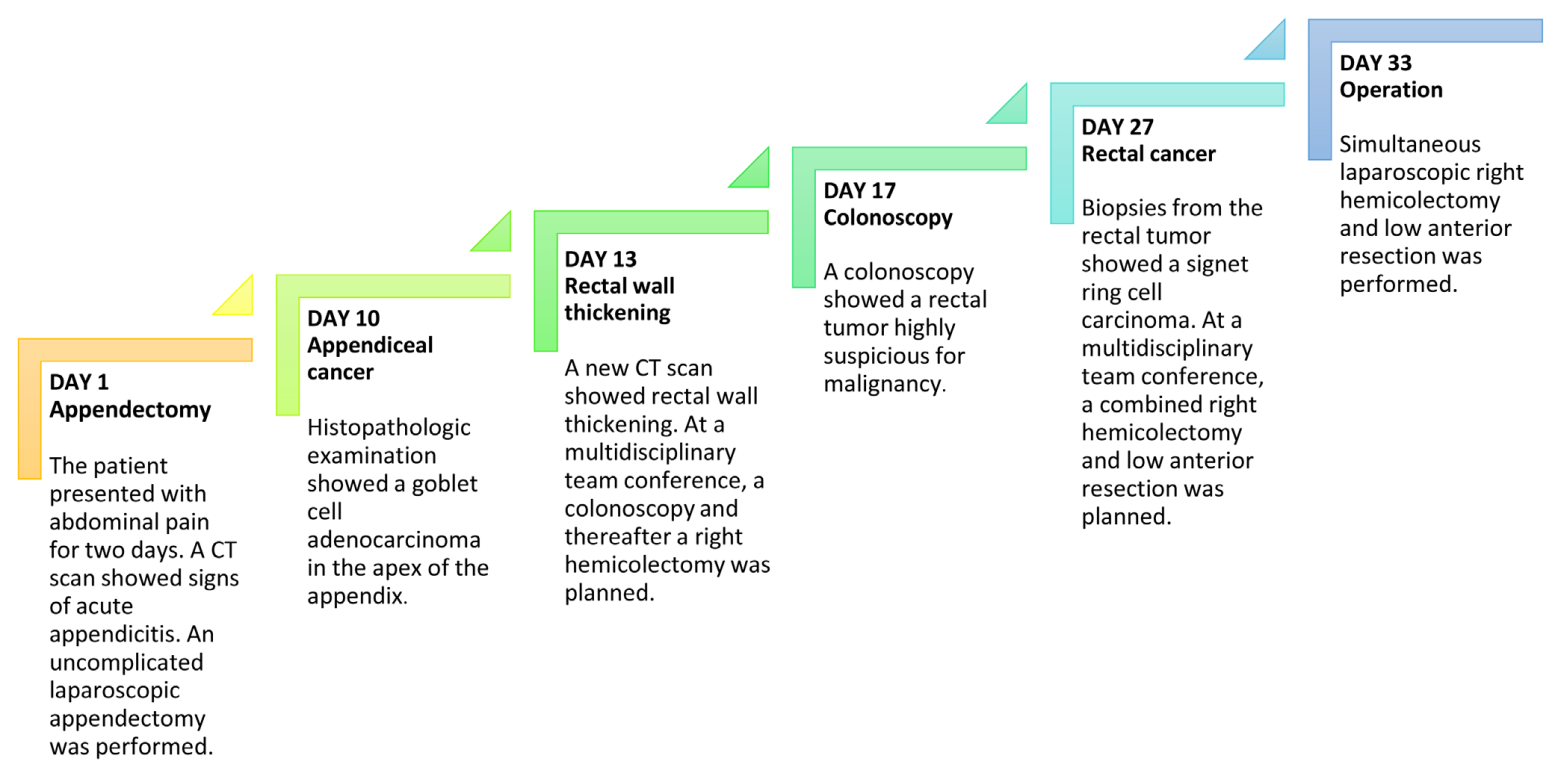
Timeline of events. CT; computed tomography.

A 58-year-old Caucasian man with no prior medical or surgical history was admitted to the emergency department after referral from his general practitioner. The patient complained of constant diffuse abdominal pain for two days with exacerbation upon movement. He had had fever for one day. His stool was normal without blood and there was no nausea or vomiting. The patient had no family history of colorectal cancer. The abdominal examination revealed direct tenderness in both lower quadrants. The DRE that was performed by the newly qualified doctor on call was without palpable tumors. Vital signs were as follows: blood pressure 131/87 mmHg, pulse 78 beats/min, and temperature 37.8 °C. Laboratory blood tests showed elevated C-reactive protein (110 mg/L [normal value less than 3 mg/L]) and a normal leukocyte count (6.5 × 10 ^9^/L [normal value 4.4 to 10.5 × 10 ^9^/L]). All other blood tests were normal. An acute computed tomography (CT) scan of the abdomen showed signs of acute appendicitis. A diagnostic laparoscopy confirmed the diagnosis of nonperforated appendicitis and an uncomplicated laparoscopic appendectomy was performed. The patient was discharged the following day. The postoperative standard histopathologic examination of the appendix showed acute non-perforated appendicitis and, surprisingly, a 13 mm T1 goblet cell adenocarcinoma in the apex of the appendix with tumor-free resection margins. The patient was informed and underwent a new CT scan of the thorax and abdomen. The only new finding on the repeated CT scan was rectal wall thickening. The patient was discussed at a multidisciplinary team conference where it was decided to perform a colonoscopy due to the rectal wall thickening and thereafter a laparoscopic right hemicolectomy due to the tumor in the appendix. Prior to the colonoscopy, the endoscopist, who is an experienced colorectal surgeon, performed a DRE. The endoscopist palpated the distal part of a tumor in the anterior wall of the rectum seven centimeters from the anal opening. The colonoscopy showed a tumor highly suspicious for malignancy with a central depression of 25 mm ( Figure 1). It was classified as National Institute for Clinical Excellence (NICE) type 3. A magnetic resonance imaging (MRI) scan staged the tumor as a T2 without metastasis to lymph nodes or distant metastasis ( Figure 2). The histopathologic examination of the rectal lesion showed a primary signet ring cell carcinoma that was histologically distinct from the appendiceal tumor. Hence, the patient had two primary synchronous tumors. Eventually, the patient was treated with simultaneous laparoscopic right hemicolectomy and low anterior resection. A protective loop ileostomy was also performed. Figure 3 presents a timeline of the events. Following surgery, the pTNM (pathological tumor-node-metastasis) classification of the appendiceal adenocarcinoma was T1N0M0 and for the rectal carcinoma T2N1M0. The postoperative course was complicated by an anastomotic leakage of the colorectal anastomosis. The leakage was treated with endoscopic vacuum-assisted closure. After the postoperative infection had been successfully managed, the patient received adjuvant chemotherapy for the rectal carcinoma. The decision to treat the patient with adjuvant chemotherapy was multifactorial: N1 staging, presence of tumor satellites, anastomotic leakage, and signet ring cell carcinoma with tumor tissue near the resection margin with a tumor deposit located 1.5 mm from the mesorectal fascia.

## Discussion

This case illustrates the importance of a full colorectal workup in patients with a primary tumor in the appendix. This is supported by current clinical guidelines. The American Society of Colon and Rectal Surgeons’ clinical guideline strongly recommends that a colonoscopy should be performed to exclude synchronous colorectal lesions in patients with appendiceal neoplasms, and also strongly recommends a complete physical examination including a DRE ^3^.

In this case, the patient’s rectal tumor was missed during the DRE performed by a newly qualified doctor in the emergency department. Studies have shown that newly qualified doctors lack confidence in performing a DRE, and the DRE is rarely repeated or supervised by a senior doctor ^11, 12^. Also, clinicians often avoid performing a DRE ^12, 13^. The DRE is a cost-efficient and quick procedure to assess and identify numerous conditions in both sexes, e.g. anal fissures, skin tags, pilonidal sinuses, anal fistulas, rectal prolapse, anal warts, skin diseases (e.g. dermatitis), anorectal tumors, gastrointestinal tract bleeding, abscesses, hemorrhoids, sphincter function, constipation, fecal impaction, prostatic hypertrophy, prostate tumors, prostatitis, pelvic inflammatory disease, pelvic floor prolapse (e.g. rectocele), and pelvic floor dyssynergia ^12, 14– 16^. Hence, the importance of a sufficient DRE should not be underestimated.

Medical school is the foundation where students learn the necessary basic skills enabling them to practice medicine when becoming qualified doctors. Worryingly, senior medical students lack training in performing a DRE and up to 44% have never performed the examination on a human subject when graduating ^17, 18^. A randomized controlled study found that medical students that had practiced DRE on phantoms and human volunteers had an increased confidence in performing and trusting the results of the examination compared with students who only practiced on phantoms ^19^. Like many other practical clinical skills, the DRE has a learning curve. A questionnaire survey concluded that the more adequate the training in performing a DRE, the more confident the examiner was in making a diagnosis using the DRE ^12^. One study comparing novice to more experienced examiners on specially designed simulators found that the latter had a significantly higher detection rate for both prostate and rectal anomalies ^9^. This highlights the importance of sufficient training and experience in DRE. When assessing a patient with abdominal or urogenital complaints, the abdominal examination is a core clinical skill. Many clinicians will experience that the abdominal examination is frequently repeated by a fellow and more experienced clinician, but this is not the case with the DRE ^11, 18, 20^. As both the abdominal examination and the DRE are standard and important clinical tools of screening in patients admitted to the emergency department, one could question why the abdominal examination is often supervised or repeated while the DRE is not. Nonetheless, the case presented here highlights the need for improved focus on the DRE in medical training.

A strength of this case is that we have a very detailed report of the patient’s history from his first contact to his general practitioner to several months after surgery. Also, we have full and detailed reports of the histopathologic examinations as well as both images and detailed reports of the CT scans, the MRI scan, and the colonoscopy. A limitation to this case is that we do not have information on the approximate total number of DREs performed by the newly qualified doctor initially admitting the patient to the hospital making it difficult to evaluate the doctor’s experience in performing the procedure.

## Conclusion

In this case report, we present a patient with primary synchronous cancers in the appendix and rectum. The case underlines the importance of the DRE as a quick, inexpensive, and useful skill in the everyday clinic. Patients with primary appendiceal neoplasms should have a full colorectal workup and there should be an improved focus on teaching and training the DRE as it improves confidence and diagnostic accuracy of the procedure.

## Data availability 

All data underlying the results are available as part of the article and no additional source data are available.

## Consent

Written informed consent for publication of clinical details and clinical images was obtained from the patient.
