# Supplementary material for: Case Report: The importance of examining colon and rectum in patients with appendiceal cancer
Source: F1000Res. 2022 Mar 3;10:152. Originally published 2021 Feb 26. [Version 2] doi: 10.12688/f1000research.50909.2 (PMC8897609; doi:10.12688/f1000research.50909.2)
Supplement: Peer review history

# Peer review: Reviewer response for version 2

**DOI:** 10.5256/f1000research.121500.r126142

**Published:** 2022-03-17

**Authors:** Cemil Yüksel

**Affiliations:** 1 Department of Surgical Oncology, University of Health Science, Ankara Abdurrahman Yurtaslan Oncology Training and Research Hospital, Ankara, Turkey

Accept.

Are enough details provided of any physical examination and diagnostic tests, treatment given and outcomes?

Partly

Is the case presented with sufficient detail to be useful for other practitioners?

Partly

Is sufficient discussion included of the importance of the findings and their relevance to future understanding of disease processes, diagnosis or treatment?

Partly

Is the background of the case’s history and progression described in sufficient detail?

Yes

Reviewer Expertise:

NA

I confirm that I have read this submission and believe that I have an appropriate level of expertise to confirm that it is of an acceptable scientific standard.

# Peer review: Reviewer response for version 2

**DOI:** 10.5256/f1000research.121500.r126141

**Published:** 2022-03-04

**Authors:** Susanne Merkel

**Affiliations:** 1 Department of Surgery, Friedrich-Alexander-University Erlangen, Erlangen, Germany

I am completely satisfied with the improvements.

Are enough details provided of any physical examination and diagnostic tests, treatment given and outcomes?

Partly

Is the case presented with sufficient detail to be useful for other practitioners?

Yes

Is sufficient discussion included of the importance of the findings and their relevance to future understanding of disease processes, diagnosis or treatment?

Yes

Is the background of the case’s history and progression described in sufficient detail?

Yes

Reviewer Expertise:

Surgical oncology

I confirm that I have read this submission and believe that I have an appropriate level of expertise to confirm that it is of an acceptable scientific standard.

# Peer review: Reviewer response for version 1

**DOI:** 10.5256/f1000research.54002.r91612

**Published:** 2021-09-09

**Authors:** Wilhelm Graf

**Affiliations:** 1 Department of Surgical Sciences, Uppsala University, Uppsala, Sweden

Thanks for letting me review this case report. My comments are as follows:

1. In the introduction it is stated that these tumors might present as bowel obstruction but in reality this is not true since the appendix is not in the GI passage.
2. The MTD conference should be added to the timeline figure.
3. Please state the body temperature, pulse and blood pressure.
4. Give more details of the MRI exam.
5. The listing of possible findings on DRE in the discussion could be shortened and focused just on malignant findings.

Are enough details provided of any physical examination and diagnostic tests, treatment given and outcomes?

Partly

Is the case presented with sufficient detail to be useful for other practitioners?

Yes

Is sufficient discussion included of the importance of the findings and their relevance to future understanding of disease processes, diagnosis or treatment?

Partly

Is the background of the case’s history and progression described in sufficient detail?

Yes

Reviewer Expertise:

Surgery

I confirm that I have read this submission and believe that I have an appropriate level of expertise to confirm that it is of an acceptable scientific standard, however I have significant reservations, as outlined above.

# Response

**Published:** 2022-02-17

**Authors:** Hugin Reistrup

**Affiliations:** Herlev Hospital, Denmark

Dear Wilhelm Graf,

 Thank you for your valuable comments, which we have read with great interest. We have answered your comments in a point-to-point format.

 1) In the introduction it is stated that these tumors might present as bowel obstruction but in reality this is not true since the appendix is not in the GI passage.

 Reply: We agree that this is an unlikely event, but although rare, bowel obstruction due to appendiceal neoplasm has been well described in the literature. We have also referred to a reference in the manuscript to back up this statement.

 2) The MTD conference should be added to the timeline figure.

 Reply: This is a very good point. We have now elaborated the decisions taken at the MDT conferences to the timeline figure.

 3) Please state the body temperature, pulse and blood pressure.

 Reply: We have now added this information to the “Case presentation” section.

 4) Give more details of the MRI exam.

 Reply: Unfortunately, there is no further information available on the MRI exam.

 5) The listing of possible findings on DRE in the discussion could be shortened and focused just on malignant findings.

 Reply: We fully understand your point regarding this. An important message in this study is to highlight the importance of a sufficient DRE. Also, we want to illustrate that the DRE can help diagnose a lot of different conditions, both benign and malign. Hence, we have chosen to make a comprehensive list of these conditions to underline this important point to the reader. As this case also has an educational aim, we are convinced that it is important to show the vast diagnostic benefits of this simple exam.

 We hope that you find the comments and additions above to the manuscript sufficient.

# Peer review: Reviewer response for version 1

**DOI:** 10.5256/f1000research.54002.r91604

**Published:** 2021-08-24

**Authors:** Cemil Yüksel

**Affiliations:** 1 Department of Surgical Oncology, University of Health Science, Ankara Abdurrahman Yurtaslan Oncology Training and Research Hospital, Ankara, Turkey

First of all, I would like to thank the authors for tackling a good topic. One of the most important topics in surgical training is rectal examination and it can sometimes be overlooked.

 Some suggestions for the authors:

1. Are the tumor markers of the patient high? I think some blood values ​​can be added to the article, only CRP is given.
2. Was there any peritoneal spread in the surgical findings? It is more likely to be seen in appendiceal tumors.
3. Colonoscopic view could have been better.
4. The tumoral lesion in colonoscopy could have been shown better.
5. Why was neoadjuvant treatment not planned for the patient?
6. Why was a protective ileostomy not planned for the patient? Because ileostomy should be performed for anastomosis safety in lower rectal tumors.
7. Has thoracic tomography been applied?
8. The postoperative condition of the patient has not been mentioned much. When did oral intake start?
9. The patient's family history was not mentioned.
10. Pathology results are not fully mentioned. Surgical margins? Number of lymph nodes removed? Metastasis?

As a result, the article has shortcomings in terms of surgery. However, the subject mentioned is important.

Are enough details provided of any physical examination and diagnostic tests, treatment given and outcomes?

Partly

Is the case presented with sufficient detail to be useful for other practitioners?

Partly

Is sufficient discussion included of the importance of the findings and their relevance to future understanding of disease processes, diagnosis or treatment?

Partly

Is the background of the case’s history and progression described in sufficient detail?

Yes

Reviewer Expertise:

Surgical Oncology, Gastric cancer, Colorectal cancer, Cytoreductive surgery, HİPEC

I confirm that I have read this submission and believe that I have an appropriate level of expertise to confirm that it is of an acceptable scientific standard, however I have significant reservations, as outlined above.

# Response

**Published:** 2022-02-17

**Authors:** Hugin Reistrup

**Affiliations:** Herlev Hospital, Denmark

Dear Cemil Yüksel,

 Thank you for your valuable comments, which we have read with great interest. We have answered your comments in a point-to-point format.

 1) Are the tumor markers of the patient high? I think some blood values ​​can be added to the article, only CRP is given.

 Reply: Unfortunately, we do not have tumor markers for this patient. In Denmark, tumor markers are not taken routinely when performing an uncomplicated appendectomy, and as soon as the appendiceal adenocarcinoma was diagnosed, a diagnostic colonoscopy was performed shortly afterwards. We agree that further blood samples would add value to the case and we therefore have added the leukocyte count to the “Case presentation” section.

 2) Was there any peritoneal spread in the surgical findings? It is more likely to be seen in appendiceal tumors.

 Reply: This is an interesting point, but no, there was no peritoneal spread. As stated in the manuscript, the laparoscopic appendectomy was uncomplicated and there were no other abnormalities described during the laparoscopy. To further elaborate the laparoscopic findings, we have now added that the patient had a nonperforated appendicitis in the “Case presentation” section.

 3) Colonoscopic view could have been better.

 Reply: We completely agree that the photo (figure 1) from the colonoscopy could have been of a better quality. Unfortunately, this was the best colonoscopic view available. Still, we are convinced that it does add value to the case.

 4) The tumoral lesion in colonoscopy could have been shown better.

 Reply: Here, we presume that you are referring to the same photo as mentioned in comment no. 3. Therefore, we would like to refer to our response to that comment.

 5) Why was neoadjuvant treatment not planned for the patient?

 Reply: Unfortunately, this information is not supplied in the patient’s journal. But according to Danish guidelines on neoadjuvant treatment of resectable rectal cancers, rectal tumors classified as T1 tumors are not treated with preoperative chemotherapy.

 6) Why was a protective ileostomy not planned for the patient? Because ileostomy should be performed for anastomosis safety in lower rectal tumors.

 Reply: This is a very good point. A protective loop ileostomy was actually performed. We have added this information to the “Case presentation” section.

 7) Has thoracic tomography been applied?

 Reply: Yes, as mentioned in the “Case presentation” section, a CT thorax and abdomen was performed. *“…* *The patient was informed and underwent a new CT scan of the thorax and abdomen.*”

 8) The postoperative condition of the patient has not been mentioned much. When did oral intake start?

 Reply: The patient started oral intake the day after surgery, which is standard in Denmark following this type of surgery. We have deliberately chosen to omit details such as these from the manuscript to keep the manuscript short and concise.

 9) The patient's family history was not mentioned.

 Reply: As mentioned in the “Case presentation” section, there was no family history of colorectal cancer. *“…* *The patient had no family history of colorectal cancer* *”*.

 10) Pathology results are not fully mentioned. Surgical margins? Number of lymph nodes removed? Metastasis?

 Reply: We fully agree that this has not been elaborated sufficiently in the case, hence, we have added the following to the “Case presentation” section.

 - “…Following surgery, the pTNM (pathological tumor-node-metastasis) classification for the appendiceal adenocarcinoma was T1N0M0 and for the rectal carcinoma T2N1M0”

 - “…After the postoperative infection had been successfully managed, the patient received adjuvant chemotherapy for the rectal carcinoma. The decision to treat the patient with adjuvant chemotherapy was multifactorial: N1 staging, presence of tumor satellites, anastomotic leakage, and signet ring cell carcinoma with tumor tissue near the resection margin with a tumor deposit located 1.5 mm from the mesorectal fascia.”

 We hope that you find the comments and additions above to the manuscript sufficient.

# Peer review: Reviewer response for version 1

**DOI:** 10.5256/f1000research.54002.r87541

**Published:** 2021-06-28

**Authors:** Susanne Merkel

**Affiliations:** 1 Department of Surgery, Friedrich-Alexander-University Erlangen, Erlangen, Germany

The authors present an interesting case report of a common condition (acute appendicitis) with an incidental finding of increasing incidence (primary adenocarcinoma of the appendix). They emphasize the importance of rectal digital examination.

 The case report is well written according to the CARE guidelines. However, I miss the pTNM classification of appendiceal carcinoma after appendectomy plus hemicolectomy and the pTNM classification of rectal carcinoma. I also miss the rationale for the indication for adjuvant chemotherapy: Synchronous multiple carcinomas? Signet ring cell carcinoma? Anastomotic leak? Lymph node metastases?

Are enough details provided of any physical examination and diagnostic tests, treatment given and outcomes?

Partly

Is the case presented with sufficient detail to be useful for other practitioners?

Yes

Is sufficient discussion included of the importance of the findings and their relevance to future understanding of disease processes, diagnosis or treatment?

Yes

Is the background of the case’s history and progression described in sufficient detail?

Yes

Reviewer Expertise:

Surgical oncology

I confirm that I have read this submission and believe that I have an appropriate level of expertise to confirm that it is of an acceptable scientific standard, however I have significant reservations, as outlined above.

# Response

**Published:** 2022-02-17

**Authors:** Hugin Reistrup

**Affiliations:** Herlev Hospital, Denmark

Dear Susanne Merkel,

 Thank you for your valuable comments, which we have read with great interest. We have answered your comments in a point-to-point format.

 1) The case report is well written according to the CARE guidelines. However, I miss the pTNM classification of appendiceal carcinoma after appendectomy plus hemicolectomy and the pTNM classification of rectal carcinoma.

 Reply: We agree that the pTNM classification would be of value to the present case. Therefore, we have added this to both the description of the appendiceal adenocarcinoma after appendectomy plus hemicolectomy (T1N0M0) and rectal carcinoma (T2N1M0) in the “Case presentation” section.

 2) I also miss the rationale for the indication for adjuvant chemotherapy: Synchronous multiple carcinomas? Signet ring cell carcinoma? Anastomotic leak? Lymph node metastases?

 Reply: We agree that the reason for choosing to treat the patient with adjuvant chemotherapy should be elaborated. The patient received neoadjuvant chemotherapy for the rectal carcinoma (signet cell ring carcinoma). The appendiceal carcinoma (goblet cell adenocarcinoma) did not require further treatment postoperatively. The oncologists based their decision on adjuvant chemotherapy on the following: N+, tumor satellite, anastomotic leakage, and signet ring cell carcinoma with tumor tissue near the resection margin with a tumor deposit located 1.5 mm from the mesorectal fascia. We have added these details to the “Case presentation” section.

 We hope that you find the additions above to the manuscript sufficient.
